# An egalitarian approach for sharing the cost of a spanning tree

**DOI:** 10.1371/journal.pone.0236058

**Published:** 2020-07-30

**Authors:** José-Manuel Giménez-Gómez, Josep E. Peris, Begoña Subiza

**Affiliations:** 1 Departament d’Economia and ECO-SOS, Universitat Rovira i Virgili, Reus, Spain; 2 Mètodes Quantitatius i Teoria Econòmica and IUDESP, Universitat d’Alacant, Alacant, Spain; Universidad de Murcia, SPAIN

## Abstract

A *minimum cost spanning tree* problem analyzes the way to efficiently connect individuals to a source. Hence the question is how to fairly allocate the total cost among these agents. Our approach, reinterpreting the spanning tree cost allocation as a claims problem defines a simple way to allocate the optimal cost with two main criteria: (1) each individual only pays attention to a few connection costs (the total cost of the optimal network and the cost of connecting himself to the source); and (2) an egalitarian criteria is used to share costs. Then, using claims rules, we define an egalitarian solution so that the total cost is allocated as *equally* as possible. We show that this solutions could propose allocations outside the core, a counter-intuitive fact whenever cooperation is necessary. Then we propose a modification to get a core selection, obtaining in this case an alternative interpretation of the *Folk* solution.

## Introduction

We consider a situation in which some individuals, located at different places, want to be connected to a source in order to obtain a good or a service. Each link connecting any two individuals, or connecting each individual to the source, has a specific fixed cost. This situation is known as the *minimum cost spanning tree* problem (hereafter, the *mcst* problem) and it is used to analyze different actual issues, such as telephone, cable TV or water supply networks.

There are several methods for obtaining a way of connecting agents to the source so that *the total cost of the selected network is minimum*. Once the minimum cost network is built, several solutions have been proposed to allocate the cost among the individuals, such as *Bird rule* [1], *Kar* [2], *Folk* [3, 4], *Cycle-complete* [5], a family of *strict responsive* rules [6], or the class of egalitarian Shapley value solutions [7]. Some of these solutions take into account the cost of each link in the network, so all the costs are relevant in order to set the final allocation of the optimal cost, although most of them would never be used. Contrary to this trend, we define a model in which only a few costs in the network are considered.

In doing so, our main assumption is that individuals are not worried about costs of links not being used. They have a *local* vision of the network and only a few information is relevant to each individual. At this point, it is important to remark that cooperation is needed to build the efficient network. That is, if some individual does not agree with the allocation of the cost, this individual could connect to the source on his own and the cheapest network is not built.

**Remark 1** *Actual situations reveal that agents do not necessarily agree on how to distribute this cost, and in those cases the social optimum is not implemented. Hence, a more expensive network is built (for an example, see* [8]; *see also* [9] *for a discussion about individual and social optimality)*.

Then, we follow a *reductionist* approach in which some information is not used, or simplified, when obtaining the cost sharing of a *mcst* problem. Specifically, we suppose that individuals are only concerned about two particular costs:

1. Their minimum cost to connect to the source (either directly or throughout other individuals).
2. The total cost of the optimal network.

If there is no cooperation, each agent will connect to the source by himself and the total cost of the network could be much higher than the optimal one (the minimum cost spanning tree).

Our second key point is the use of an *egalitarian* criterion. Taking into account that no one should pay more than the cost of connecting to the source by himself (*individual rationality*), our approach will allocate the cost based on an equal distribution of the benefit of cooperation. This approach leads us to solve the allocation problem by using claims rules. Let us observe two problems that illustrate our idea.

(A) *Consider a set of three houses in a row (at the same distance each one from the other). A water supply ω is located at one end of the row. The cost of each link between two adjacent houses is 1 monetary unit. The nearest house to the supply may connect directly with a cost of 10 units; the second house has a direct cost of 11 units; and the cost of directly connecting the farthest house is 12 units. The total (minimum) cost of connecting the three houses to the water supply is 12 units. If each individual connects directly to the source, the total cost is 33 units.*

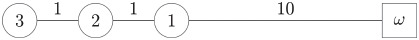
  In this situation, the cooperation provides a benefit (savings) of 21 monetary units. The question is how to share this benefit. Note that individual 1 is in a “better position” and a lower payment of this individual would be reasonable. If the benefit of cooperation is equally shared, each agent obtains a return of 7 unitary units, paying, respectively, 3, 4 and 5 monetary units. But an egalitarian cost sharing will propose an allocation of 4 units for each individual.
(B) *Consider a similar situation, but now individual 1 is closer to the source, whereas individuals 2 and 3 remain at the same location and the costs vary accordingly (the new situation is as depicted in the following graph):*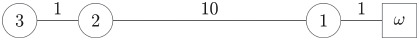  As in the previous case, the total (minimum) cost of connecting the three houses to the water supply is 12 units. But the situation is quite different and now cooperation provides a benefit of 12 monetary units. An equal sharing of the benefits originates a negative allocation to individual 1; that is, he obtains a net benefit from his participation in the network. On the other hand, observe that in the new situation an equal allocation of the cost is not admissible for the first individual, since he may connect to the source with a cost of 1 unit, instead of paying 4 units.

As aforementioned, our main criterion in allocating the cost of the optimal tree is that of *egalitarian sharing*, which is one of the main criterion supported in the literature. In [10] the egalitarian method appear, jointly with the proportional, as the most important (and simple) ways of sharing a joint cost or benefit.

The rest of the paper is organized as follows. Next Section presents the formal minimum cost spanning tree problem. Then, we relate minimum cost spanning tree problems with claims problems, introduce the egalitarian solution concept and analyze its properties. We show that, in general, our proposal may lie outside the core in some situations. In our last Section we propose a modification fulfilling core stability, and we obtain a new interpretation of the *Folk* solution. Some final comments close the paper.

## Preliminaries: Minimum cost spanning tree problem

A *mcst* problem involves a finite set of individuals, *N* = {1, 2, …, *n*}, who want to be connected to a *source* *ω*. Let *N*_*ω*_ = *N*∪{*ω*}. The agents are connected by edges and for *i* ≠ *j*, $c_{ij}\in R_{+}$ represents the cost of the edge *e*_*ij*_ connecting agents *i*, *j* ∈ *N*. Following the notation in [2], *c*_*ii*_ represents the cost of the edge connecting agent *i* ∈ *N* to the source *ω*. Let **C** = [*c*_*ij*_]_*n*×*n*_ the *n* × *n* symmetric cost matrix. The *mcst* problem is represented by the pair (*N*_*ω*_, **C**). We denote by $N_{n}$ the set of all *mcst* problems with *n* individuals.

A *spanning tree* over $\left(N_{\omega },C\right)\in N_{n}$ is an undirected graph *p* with no cycles, which connects all elements of *N*_*ω*_. We can identify a spanning tree with a function *p*: *N* → *N*_*ω*_ so that *p*(*i*) is the agent (or the source) to whom *i* connects in his path to the source, and defines the edges $e_{i}^{p}=\left(i,p\left(i\right)\right).$ In a spanning tree each agent is (directly or indirectly) connected to the source *ω*. Moreover, given a spanning tree *p*, there is a single path from any *i* ∈ *N* to the source *ω*, given by the edges (*i*, *p*(*i*)), (*p*(*i*), *p*^2^(*i*)), …, (*p*^*t*−1^(*i*), *p*^*t*^(*i*) = *ω*), for some integer *t* < *n*. Let us denote by $S(N_{\omega })$ the set of all spanning trees in the problem (*N*_*ω*_, **C**). The cost of building a spanning tree $p\in S(N_{\omega })$ is the sum of the costs of all the edges in this tree; that is (with some abuse of notation, when *p*(*i*) = *ω*, *c*_*ip*(*i*)_ = *c*_*ii*_)

$$\begin{array}{c} C_{p}=\sum_{i=1}^{n}c_{ip(i)}=\sum_{i=1}^{n}c(e_{i}^{p}) \end{array}$$

Given a spanning tree $p\in S(N_{\omega })$, we denote by *p*(*i*, *j*) the set of edges in the (unique) path in *p* joining *i* and *j*.

Prim [11] provides an algorithm that solves the problem of connecting all the agents to the source at the *minimum cost*. This method has *n* steps, as much as the number of individuals in the network. First, it connects to the source the agent *i* with smallest cost to the source, *c*_*ii*_ ≤ *c*_*jj*_, for all *j* ∈ *N*. In case that more than one agent fulfills this condition, any of them can be selected. In the second step, an agent in *N*\{*i*} with the smallest cost either to the source or to agent *i*, who is already connected, is selected and this connection is used. The process continues until all agents are connected, at each step connecting an agent still not connected to a connected agent or to the source. We denote by *m* a tree with minimum cost and by *C*_*m*_ its cost. That is, for all spanning tree *p*,

$$\begin{array}{c} C_{m}=\sum_{i=1}^{n}c_{im(i)}\leq C_{p}=\sum_{i=1}^{n}c_{ip(i)}. \end{array}$$

Once a network is built, an important issue is how to allocate the associated cost among the agents. A *cost sharing rule* for *mcst* problems is a function $\alpha :N_{n}\to R^{n}$ that proposes for any *mcst* problem (*N*_*ω*_, **C**) an allocation $\left(\alpha_{1},\alpha_{2},\ldots ,\alpha_{n}\right)\in R^{n},$ such that

$$\begin{array}{c} \sum_{i=1}^{n}\alpha_{i}=C_{m}. \end{array}$$

**Remark 2** *In some contexts the non-negativity of the cost α*_*i*_ *allocated to each individual is required. This question is related to the assumption of property or non-property rights on the locations that individuals occupy (see, for instance*, [6] *for a discussion). In the second case, non-property rights approach, the allocations must be necessarily non-negative. In what follows we will consider the non-property rights approach*.

Bird [1] proposes a cost allocation so that each individual pays the cost of the edge he directly uses to be connected in the minimum cost spanning tree. In case there are several networks providing the (same) minimum cost, the *Bird* solution allocates to each individual the average of the cost of the connections he uses in these networks. Since then, several authors have proposed other solution concepts in the *mcst* literature: for instance, [2, 3, 4, 5, 6, 12], etc. (see [13] for definitions and a comparative analysis of most of these solutions).

Some of these solutions take all the possible connections in the graph into account, although most of these connections are not used in the optimal tree. However, other solutions use a *reductionist approach* and they are obtained only considering some of the connection costs. Specifically, the *Bird* solution only considers the cost of the link each individual uses in the optimal network while the costs of other edges are ignored. The *Folk* and *Cycle-complete* solutions also take a reductionist approach. As the reductionist approach ignores some of the available information it reduces the parameters of the problem (and the computational complexity).

In order to analyze the cost that any individual would incur without cooperation, we introduce the notion of *indirect* cost.

**Definition 1** *Given a mcst problem* (*N*_*ω*_, **C**) *the **indirect** cost to connect individual i* ∈ *N to the source ω is*

$$\begin{array}{c} c_{ii}^{*}=\min_{p\in S(N_{\omega })}\{\sum c(e)\;e\in p(i,\omega )\} \end{array}$$

In this context, demanding that the maximum cost to be allocated to any individual cannot exceed his (indirect) cost to the source (*individual rationality*) is a compulsory requirement because, in other case, the individual would be better off acting by himself and would not cooperate in building the optimal network.

**Definition 2** *A cost allocation α* = (*α*_1_, *α*_2_, …, *α*_*n*_) *of the minimum cost C*_*m*_ *in a cst problem* (*N*_*ω*_, **C**), *is **individually rational** if for all i* ∈ *N*, $\alpha_{i}\leq c_{ii}^{*}.$

Whenever cooperation is necessary, as in *mcst* situations, the literature on cost sharing singles out core stability as the key property of any allocation rule: no coalition of agents should be charged more than their cost of connecting to the source. Then, given a coalition *S* ⊆ *N*, the *stand alone* cost for this coalition to be connected to the source (in our *non-property rights* model) is:

$$\begin{array}{c} v\left(S\right)=\min\{C_{m}\left(T\right):\;S\subseteq T\subseteq N\} \end{array}$$

where *C*_*m*_(*T*) denotes the cost of the optimal tree connecting coalition *T* to the source. Note that for any *i* ∈ *N*, $v(\{i\})=c_{ii}^{*}.$

**Remark 3** *It is important to note that the cost function C*_*m*_(*S*), *S* ⊆ *N*, *is not monotonic since the addition of some agents may reduce the cost of the coalition. As we follow the non-property rights approach, any coalition S might use locations of individuals outside S to build their minimum cost spanning tree. So*, *v*(*S*) *represents the minimum cost of connecting all individuals in S to the source ω*, *possibly using (and paying for) connections of individuals outside S*. *The stand alone cost function v*(*S*) *is monotonic*.

**Definition 3** *A cost allocation α* = (*α*_1_, *α*_2_, …, *α*_*n*_) *of the minimum cost C*_*m*_ *in a mcst problem* (*N*_*ω*_, **C**) *is a **core selection** if for all S* ⊆ *N*, *S* ≠ ∅, $\sum_{i\in S}\alpha_{i}\leq v(S)$.

## Egalitarian cost sharing

We propose an egalitarian treatment of the agents. So, a first attempt to allocate the cost of the optimal network, *C*_*m*_ is to divide it equally among the individuals: $\alpha_{i}=\frac{1}{n}C_{m}$, *i* = 1, 2, …, *n*. However, as Problem (B) depicts, the equal division may be not individually rational: agent 1 is allocated 4 monetary units, whereas his cost to the source is $c_{11}^{*}=1$ monetary unit.

Individual rationality provides a way to address the problem of allocating the optimal cost in a *mcst* problem by transforming it into a surplus sharing problem:

- First, each agent pays his (indirect) cost to connect the source $c_{ii}^{*}$. So, the individuals jointly contribute with the amount $C^{*}=\sum_{i=1}^{n}c_{ii}^{*}$ to build a network.
- Then, the efficient tree may be built, with cooperation, at a cost *C*_*m*_ ≤ C* and there is a benefit from cooperation given by *B* = C* − *C*_*m*_.
- Any method used to share this benefit *B*, $\sum_{i=1}^{n}x_{i}=B,$ *x*_*i*_ ≥ 0, provides a *final allocation* of the cost *C*_*m*_, $\alpha_{i}=c_{ii}^{*}-x_{i}$, which is individually rational.

A possible way is to share equally the benefits obtained from cooperation:

$$\begin{array}{c} \alpha_{i}=c_{ii}^{*}-\frac{C^{*}-C_{m}}{n}\;i=1,2,\ldots ,n. \end{array}$$

However, as Problem (B) shows, equalizing benefits could end in a negative allocation for some agents, that implies that these agents get a net profit from participating in the network. As mentioned in [6] this possibility only has sense if the individuals have property rights on their location. It is noteworthy that if we want to avoid this possibility (since we are in the non-property rights approach) a *constrained equal division* should be considered: *no one obtains a benefit greater than his initial contribution*. Then the benefit of cooperation should be shared as in a claims problem in which each individual *claims all his contribution to be returned*. A *claims problem* is a situation involving *n* individuals who claim some amount *d*_*i*_, so that the aggregate demand exceeds the available endowment *B*, $\sum_{i=1}^{n}d_{i}\geq B$. A *claims rule* *φ* divides efficiently the endowment so that agents do not receive a negative amount, nor more than their claim. The following definition transforms *mcst* problems into claims problems (see [14] for a complete survey on claims problems).

**Definition 4** *To any mcst problem* (*N*_*ω*_, **C**) *with minimum cost C*_*m*_, *we associate the claims problem* (*B*, *d*), *where B* = C* − *C*_*m*_, $C^{*}=\sum_{i=1}^{n}c_{ii}^{*}$ *and* $d_{i}=c_{ii}^{*}$.

That is, each agent claims the amount he paid: his indirect cost to the source. If we use a claims rule *φ* to solve the problem (*B*, *d*), then $\alpha_{i}=c_{ii}^{*}-\varphi_{i}$ is an individually rational allocation of the cost *C*_*m*_. The claim boundedness condition, *φ*_*i*_ ≤ *d*_*i*_, implies that the allocations $\alpha_{i}=c_{ii}^{*}-\varphi_{i}$ are non-negative, which is coherent with the non-property rights approach. As we are interested in an egalitarian cost sharing, we consider the two main egalitarian claims rules: the *Constrained Equal Awards* (*CEA*) and the *Constrained Equal Losses* (*CEL*), that equalize, respectively, gains and losses satisfying the restrictions of a claims rule. Formally, given a claims problem $\left(B,d\right)\in R_{+}\times R_{+}^{n}$

$$\begin{array}{cc} CEA_{i}(B,d)=\text{min}\left\{\lambda ,d_{i}\right\} & \lambda \text{such}\;\text{that}\sum_{i\in N}CEA_{i}(B,d)=B\\ CEL_{i}(B,d)=\text{max}\left\{-\lambda +d_{i},0\right\} & \lambda \text{such}\;\text{that}\sum_{i\in N}CEL_{i}(B,d)=B \end{array}$$

**Definition 5** *Given a mcst problem* (*N*_*ω*_, **C**) *such that the cost of the optimal spanning tree is C*_*m*_, *let B* = C* − *C*_*m*_ and $d_{i}=c_{ii}^{*}$; *then, the **constrained equal costs** sharing rule assigns to each individual i* ∈ *N the amount*

$$\begin{array}{c} \alpha_{i}^{ceq}\left(N_{\omega },C\right)=c_{ii}^{*}-CEL_{i}\left(B,d\right). \end{array}$$

This sharing rule allocates the same amount to all agents, constrained to no one is allocated an amount greater than his indirect cost to the source. Example 1 compares this solution with *Bird* and *Folk* proposals in the *mcst* problems **(A)** and **(B)**.

**Example 1** *For problems **(A)*** *and **(B)*** *the following cost shares are obtained*:

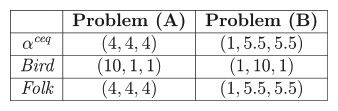


Taking the *Folk* solution as a benchmark, we analyze if its main properties are satisfied or not by our egalitarian proposal *α^ceq^*. First, we formally define these properties (see [4] and [6] for relationships and interpretations of these properties).

- A solution *α* for *mcstp* satisfies **positivity** if *α*(*N*_*ω*_, **C**)≥0, for any problem (*N*_*ω*_, **C**).
- A solution *α* for *mcst* problems satisfies **continuity** if it is a continuous function of the cost matrix **C**.
- A solution *α* for *mcstp* satisfies **symmetry** if for any problem (*N*_*ω*_, **C**) such that there are *i*, *j* ∈ *N* with *c*_*ik*_ = *c*_*jk*_, for all *k* ∈ *N*\{*i*, *j*}, *c*_*ii*_ = *c*_*jj*_, then *α*_*i*_(*N*_*ω*_, **C**) = *α*_*j*_(*N*_*ω*_, **C**).
- A solution *α* for *mcst* problems satisfies **strong cost monotonicity** if for any pair of problems (*N*_*ω*_, **C**), (*N*_*ω*_, **C**′) such that **C** ≤ **C**′, then *α*_*i*_(*N*_*ω*_, **C**)≤*α*_*i*_(*N*_*ω*_, **C**′) for all *i* ∈ *N*.
- A solution *α* for *mcst* problems satisfies **cost monotonicity** if for any pair of problems (*N*_*ω*_, **C**), (*N*_*ω*_, **C**′) such that **C** and **C**′ coincide except that $c_{ik}<c_{ik}^{\prime }$, for some *i*, *k* ∈ *N*, then *α*_*i*_(*N*_*ω*_, **C**)≤*α*_*i*_(*N*_*ω*_, **C**′).
- A solution *α* for *mcst* problems satisfies **population monotonicity** if for any problem (*N*_*ω*_, **C**), any subset *S* ⊆ *N* and any *i* ∈ *S*, *α*_*i*_(*S*_*ω*_, **C**|_*S*_)≥*α*_*i*_(*N*_*ω*_, **C**).
- A solution *α* for *mcst* problems satisfies **ranking** if for any problem (*N*_*ω*_, **C**) and *i*, *j* ∈ *N* such that *c*_*ik*_ ≤ *c*_*jk*_ for all *k* ∈ *N*\{*i*, *j*}, *c*_*ii*_ ≤ *c*_*jj*_, then *α*_*i*_(*N*_*ω*_, **C**)≤*α*_*i*_(*N*_*ω*_, **C**).
- A *mcst* problem (*N*_*ω*_, **C**) is **separable** if there are two disjoint subsets *S*∪*T* = *N*, *S*∩*T* = ∅, such that the *mcst* in *N* are union of *mcst* in each of the sub-problems, *m*(*N*_*ω*_, **C**) = *m*^1^(*S*_*ω*_, **C**|_*S*_)∪*m*^2^(*T*_*ω*_, **C**|_*T*_). A solution *α* for *mcst* problems satisfies **separability** if for any separable problem (*N*_*ω*_, **C**), *N* = *S*∪*T*, *S*∩*T* = ∅,
  $$\alpha_{i}\left(N_{\omega },C\right)=\{\begin{array}{ccc} \alpha_{i}\left(S_{\omega },C{|}_{S}\right) & \text{if} & i\in S\\ \alpha_{i}\left(T_{\omega },C{|}_{T}\right) & \text{if} & i\in T \end{array}$$
- A solution *α* for *mcst* problems satisfies **equal share of extra-costs** if for any pair of problems (*N*_*ω*_, **C**), (*N*_*ω*_, **C**′) such that:
  1. for all *i* ∈ *N*, *c*_*ii*_ = *c*_0_, $c_{ii}^{\prime }=c_{0}^{\prime },$ $c_{0}<c_{0}^{\prime }$.
  2. for all *i*, *j* ∈ *N*, *i* ≠ *j*, $c_{ij}^{\prime }=c_{ij}\leq c_{0}$
  then $\alpha_{i}\left(N_{\omega },C^{\prime }\right)=\alpha_{i}\left(N_{\omega },C\right)+\frac{c_{0}^{\prime }-c_{0}}{n}$, for all *i* ∈ *N*.
- Two *mcst* problems (*N*_*ω*_, **C**) and (*N*_*ω*_, **C**′) are **tree equivalent** if there is a tree *m* such that it is a minimum cost spanning tree for both problems, and moreover $c_{im(i)}=c_{im(i)}^{\prime }$ for all *i* ∈ *N*. A solution *α* for *mcst* problems satisfies **independence of irrelevant trees** if for any pair of tree equivalent problems (*N*_*ω*_, **C**) and (*N*_*ω*_, **C**′), then *α*(*N*_*ω*_, **C**) = *α*(*N*_*ω*_, **C**′).

**Proposition 1** *α^ceq^ fulfills positivity, continuity, cost monotonicity, strong cost monotonicity, independence of irrelevant trees, ranking, symmetry and equal share of extra-costs. It does not fulfill population monotonicity, separability, nor core stability*.

**Proof.** First, we note that our sharing rule may be written alternatively as:

$$\begin{array}{c} \alpha^{ceq}\left(N_{\omega },C\right)=CEA\left(C_{m},c^{*}\right)\;c^{*}=(c_{11}^{*},c_{22}^{*},\ldots ,c_{nn}^{*}) \end{array}$$

**(1)** *Positivity* is immediately fulfilled, since the *CEL* rule satisfies claims boundedness and the maximum amount that can be returned is what each individual has paid.

**(2)** *Continuity*.

We know that *C*_*m*_ varies continuously with **C** (see [4]), and the indirect costs, $d_{i}=c_{ii}^{*}$ are obviously a continuous function of the cost matrix. On the other hand, *CEA* is a continuous function on its arguments, which proves that *α*^*ceq*^ is *continuous*.

**(3)** *Strong cost monotonicity*. Consider a pair of problems (*N*_*ω*_, **C**), (*N*_*ω*_, **C**′) such that **C** ≤ **C**′. Then, $C_{m}\leq C_{m}^{\prime }$ and $d_{i}=c_{ii}^{*}\leq d_{i}^{\prime }={\left(c^{\prime }\right)}_{ii}^{*}$, for all *i* ∈ *N*. Since the claims rule *CEA* fulfills endowment monotonicity and claims monotonicity (see [14]), then

$$\begin{array}{c} CEA_{i}\left(C_{m},d\right)\leq CEA_{i}\left(C_{m}^{\prime },d^{\prime }\right) \end{array}$$

that implies

$$\begin{array}{c} \alpha_{i}^{ceq}\left(N_{\omega },C\right)\leq \alpha_{i}^{ceq}\left(N_{\omega },C^{\prime }\right)\;\forall i\in N. \end{array}$$

Since this property implies *cost monotonicity* and *independence of irrelevant trees* (see [4]), all three properties are fulfilled.

**(4)** *Ranking*. Given a problem (*N*_*ω*_, **C**) and *i*, *j* ∈ *N* such that *c*_*ik*_ ≤ *c*_*jk*_ for all *k* ∈ *N*, then it is obvious that $d_{i}=c_{ii}^{*}\leq d_{j}=c_{jj}^{*}$. Therefore, *CEA*_*i*_(*C*_*m*_, *d*)≤*CEA*_*j*_(*C*_*m*_, *d*) (order preservation, see [14]) and *α*^*ceq*^ fulfills *ranking*. Since *ranking* implies *symmetry* this property is also fulfilled.

## A core egalitarian proposal

The main objection to *α^ceq^*> is that, in general, it fails to be a core selection. Then, subsets of agents may have incentives to leave the grand coalition and perform their project by themselves. Next, we show some classes of *mcst* problems in which *α^ceq^*> is a core selection. Later, we will propose a modification of our solution (maintaining the egalitarian criteria) in order to achieve core selection for all *mcst* situations.

### Some *mcst* problems where *α^ceq^*> is a core-selection

In some families of *mcst* problems *α^ceq^*> always provides core allocations. We present two examples of such families.

E1) Let us consider the so-called 2 − *mcst* problems in which the connection cost between two different individuals (houses, villages, …) can only take one of two possible values: low and high cost (see, for instance, [15]; see also [16] where this class has been generalized to the so-called *simple* *mcst* problems).
  Moreover, we assume that *c*_*ij*_ = *k*_1_, *i* ≠ *j*, *c*_*ii*_ = *k*_2_, and 0 ≤ *k*_1_ ≤ *k*_2_. Note that, for this family of *mcst* problems the property *equal share of extra-costs* could be applied.
  In this case, if there are *n* individuals, then *C*_*m*_ = *k*_2_ + (*n* − 1)*k*_1_ and $d_{i}=c_{ii}^{*}=k_{2}$. As all claims are identical, then
  $$\begin{array}{c} \alpha_{i}^{ceq}=\frac{k_{2}}{n}+\frac{n-1}{n}k_{1}\;i=1,2,\ldots ,n \end{array}$$
  and, for all *S* ⊆ *N*, *v*(*S*) = *k*_2_+ *k*_1_(|*S*| − 1). Then, since *k*_1_ ≤ *k*_2_
  $$\begin{array}{c} \sum_{i\in S}\alpha_{i}^{ceq}=\sum_{i\in S}(\frac{k_{2}}{n}+\frac{n-1}{n}k_{1})=\left|S\right|(\frac{k_{2}}{n}+\frac{n-1}{n}k_{1})\leq v\left(S\right) \end{array}$$
  So the allocation provided by *α^ceq^*> belongs to the core of the cooperative game.
E2) Let us consider *linear mcst* problems: a group of individuals *N* = {1, 2, …, *n*} situated in a row (equally separated) want to connect to a source *ω*. The cost of connecting one individual with the next one is *k* monetary units. The cost of connecting individual 1 to the source is *M* monetary units. If an individual wants to connect to the source, he must do it through all its neighbors on the way towards the source and pay all costs. This is the case of Problem **(A)**.

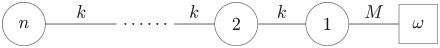
  Formally, for each *i*, *j* ∈ *N*, *i* ≠ *j*, the connection cost is *c*_*ij*_ = |*i* − *j*|*k*. For each *i* ∈ *N*, the cost to the source is *c*_*ii*_ = *M* + (*i* − 1)*k*.
  The *minimum cost spanning tree* *m* connects each individual to the next, *m*(*j*) = *j* − 1, *j* ≥ 2, and the first one with the source, *m*(1) = *ω*, with a total cost *C*_*m*_ = *M* + (*n* − 1)*k*. For each *i* ∈ *N*, $d_{i}=c_{ii}^{*}=M+\left(i-1\right)k$ and then
  $$\begin{array}{c} B=C^{*}-C_{m}=\left(n-1\right)(M+(\frac{n}{2}-1)k) \end{array}$$
  For any coalition *S* ⊆ *N*, *v*(*S*) = *M* + *k*max{*i* − 1, *i*∈*S*}. To obtain the allocation provided by *α^ceq^*> we distinguish two cases:
  1. If *M* ≥ *k*, $\alpha_{i}^{ceq}=\frac{M}{n}+\frac{n-1}{n}k,$ for all *i* ∈ *N*. Then, for all *S* ⊆ *N*
    $$\begin{array}{c} \sum_{i\in S}\alpha_{i}^{ceq}=\sum_{i\in S}(\frac{M}{n}+\frac{n-1}{n}k)=\left|S\right|(\frac{M}{n}+\frac{n-1}{n}k)\leq \end{array}$$
    $$\begin{array}{c} \leq M+\left(|S|-1\right)k\leq M+k\max\{i-1,i\in S\}=v\left(S\right). \end{array}$$
  2. If *M* < *k*, $\alpha_{1}^{ceq}=M$, $\alpha_{i}^{ceq}=k$, for all *k* ≥ 2. Then, for all *S* ⊆ *N*
    - If 1 ∈ *S*,
      $$\begin{array}{c} \sum_{i\in S}\alpha_{i}^{ceq}=M+\left(|S|-1\right)k\leq \end{array}$$
      $$\begin{array}{c} \leq M+k\max\{i-1,\;i\in S\}=v\left(S\right). \end{array}$$
    - If 1 ∉ *S*, max{*i* − 1, *i*∈*S*} ≥ |*S*| and
      $$\begin{array}{c} \sum_{i\in S}\alpha_{i}^{ceq}=\left|S\right|k\leq M+k\max\{i-1,\;i\in S\}=v\left(S\right). \end{array}$$
  So the allocation provided by *α^ceq^*> belongs to the core of the cooperative game.

### A core-egalitarian proposal

In order to obtain an egalitarian solution satisfying core stability, we express the *α^ceq^*> solution in an alternative way (see [17] to obtain an expression of the *CEA* claims rule as a minimization problem). Let A be the set of non-negative individually rational allocations in a *mcst* problem (*N*_*ω*_, **C**)

$$\begin{array}{c} A(N_{\omega },C)=\{x\in R^{n}:\sum_{i=1}^{n}x_{i}=C_{m}\;0\leq x_{i}\leq c_{ii}^{*}\;i=1,2,\ldots ,n\} \end{array}$$

then

$$\begin{array}{c} \alpha^{ceq}(N_{\omega },C)=\arg\min\{\sum_{i=1}^{n}{\left(x_{i}-\frac{C_{m}}{n}\right)}^{2}\;x\in A(N_{\omega },C)\} \end{array}$$

Note that, as the distance function is continuous and strictly convex, and set A is compact and convex, the minimization problem has always a unique solution. This expression provides us a way of obtaining a core allocation that tries to meet our egalitarian criteria, by defining:

$$\begin{array}{c} \beta^{ceq}(N_{\omega },C)=\arg\min\{\sum_{i=1}^{n}{\left(x_{i}-\frac{C_{m}}{n}\right)}^{2}\;x\in co(N_{\omega },C)\} \end{array}$$

where *co*(*N*_*ω*_, **C**) denotes the core of the cooperative game defined by the characteristic function *v*(*S*), *S* ⊆ *N*. Obviously, this proposal is the most egalitarian core allocation. On the other hand, as $co(N_{\omega },C)\subseteq A(N_{\omega },C)$, we obtain the following result:

$$\begin{array}{c} \text{If}\;\;\alpha^{ceq}(N_{\omega },C)\in co(N_{\omega },C)\;\text{then}\;\beta^{\text{ceq}}(N_{\omega },C)=\alpha^{ceq}(N_{\omega },C) \end{array}$$

In Example 2, $\alpha^{ceq}(N_{\omega },C)=(3/2,3/2,3/2,3/2)\notin co(N_{\omega },C)$, and $\beta^{ceq}$ coincides with the *Folk* solution. We show that this coincidence is always true in this kind of *mcst* problems.

**Proposition 2** *In the class of* 2 − *mcst problems* *β^ceq^* coincides with the *Folk* solution.

**Proof.** From [16] we know that in this class of problems there is a partition of the set of agents, such that in each subset the *Folk* solution proposes the same allocation to all individuals in this group (*simple* components). To simplify the proof we suppose that there are just two components in the 2 − *mcst* problem (*N*_*ω*_, **C**) (for more than two components, the reasoning follows an analogous argument):

$$\begin{array}{c} N=N_{1}\cup N_{2},\;N_{1}\cap N_{2}=\emptyset ,\;F_{i}=c_{1},\;\forall i\in N_{1},\;F_{j}=c_{2},\;\forall j\in N_{2} \end{array}$$

Let us denote by *n*_*i*_ the cardinality of the subset *N*_*i*_, *i* = 1, 2, *n* = *n*_1_+ *n*_2_ and, as usually, *C*_*m*_ is the cost of the optimal tree. Then, applying separability

$$\begin{array}{c} n_{1}c_{1}+n_{2}c_{2}=C_{m},\;n_{1}c_{1}=v\left(N_{1}\right),\;n_{2}c_{2}=v\left(N_{2}\right)\;v\left(N_{1}\right)+v\left(N_{2}\right)=v\left(N\right) \end{array}$$

Moreover, we know that this allocation is in the core of the monotonic cooperative game. On the other hand, after reordering the agents, we can write

$$\begin{array}{c} \beta^{ceq}(N_{\omega },C)=(x,y)\;x=(x_{1},x_{2},\ldots ,x_{n_{1}})\;y=(y_{1},y_{2},\ldots ,y_{n_{2}}) \end{array}$$

such that (*x*, *y*) minimizes

$$\begin{array}{c} \sum_{i=1}^{n_{1}}{\left(x_{i}-\frac{C_{m}}{n}\right)}^{2}+\sum_{j=1}^{n_{2}}{\left(y_{j}-\frac{C_{m}}{n}\right)}^{2}\;(x,y)\in co(N_{\omega },C) \end{array}$$

Then, by noticing that

$$\begin{array}{c} \sum_{i=1}^{n_{1}}x_{i}=v\left(N_{1}\right)\;\sum_{j=1}^{n_{2}}y_{j}=v\left(N_{2}\right) \end{array}$$

the function minimizes when all components *x*_*i*_ are identical for all *i* ∈ *N*_1_, and *y*_*j*_ are identical for all *j* ∈ *N*_2_. Then,

$$\begin{array}{c} x_{i}=\frac{v(N_{1})}{n_{1}}=c_{1}=F_{i},\;i\in N_{1}\;y_{j}=\frac{v(N_{2})}{n_{2}}=c_{2}=F_{j},\;j\in N_{2} \end{array}$$

and both solutions coincide.

### A piece-wise linear extension

If we denote by $C_{n}$ the set of all cost matrices involving *n* individuals, **C**_*n* × *n*_, and by $C_{n}^{b}$ the set of *elementary cost matrices*, i.e., *c*_*ij*_ ∈ {0, 1} for all *i*, *j* = 1, 2, …, *n*, we know (see [6]) that there is a basis

$$\begin{array}{c} C^{1},\;C^{2},\;\ldots ,\;C^{p}\in C_{n}^{b}\;p=\frac{n(n+1)}{2} \end{array}$$

such that any cost matrix $C\in C_{n}$ can be expressed as

$$\begin{array}{c} C=\sum_{k=1}^{p}\lambda_{k}\;C^{k}\;\lambda_{k}\in R \end{array}$$

Then, given a *mcst* solution *ψ*^*b*^ defined only for elementary cost matrices (a *partial* solution), its *piece-wise linear extension* *ψ* is defined by

$$\begin{array}{c} \psi (N_{\omega },C)=\sum_{k=1}^{p}\lambda_{k}\;\psi^{b}(N_{\omega },C^{k}) \end{array}$$

As proved in [6], piece-wise linear solutions have the advantage that many normative properties automatically extend from elementary to arbitrary cost matrices. In particular, they show that this is the case with the properties of *ranking*, *cost monotonicity*, *polynomial complexity*, *population monotonicity* and *positivity*. Then, we can define the piece-wise linear extension of the *β^ceq^* solution defined only on elementary problems:

$$\begin{array}{c} ϒ(N_{\omega },C)=\sum_{k=1}^{p}\lambda_{k}\;\beta^{ceq}(N_{\omega },C^{k}) \end{array}$$

As an immediate consequence of Proposition 2 (elementary problems are a particular case of 2 − *mcst* problems) we obtain that this extension coincides with the *Folk* solution.

**Corollary 1** *For any mcst problem* (*N*_*ω*_, **C**), *Υ*(*N*_*ω*_, **C**) = *F*(*N*_*ω*_, **C**).

Then, the *Folk* solution appears as the piece-wise linear extension of *β^ceq^*, that picks the most egalitarian allocation in the core associated to the *mcst* problem, showing in this way an alternative interpretation to this solution.

### Some further comments

In [18, 19] it is shown that for elementary *mcst* problems, the *Folk* solution is the permutation-weighted average of the extreme points of the non-property rights game defined by *v*(*S*). Then, for any elementary *mcst* problem (*N*_*ω*_, **C**), as a consequence of Proposition 2 we obtain

$$\begin{array}{c} \beta^{ceq}(N_{\omega },C)=\sum_{k=1}^{K}\frac{1}{n!}{\bar{y}}^{k}(N_{\omega },C) \end{array}$$ (1)

where ${\bar{y}}^{k}(N_{\omega },C)$, *k* = 1, 2, …, *K*, denote the extreme points of the core of the cooperative game defined by *v*(*S*) (see [18]). Moreover, in this class of *mcst* problems, *β^ceq^* also coincides with the *nucleolus* (see [19]), showing the close relationships between the egalitarian and nucleolus concepts. We could define a non-piecewise linear extension of *β^ceq^* by using Eq (1).

**(5)** *Equal share of extra-costs*. If we consider two problems (*N*_*ω*_, **C**), (*N*_*ω*_, **C**′) such that:

1. for all *i* ∈ *N*, *c*_*ii*_ = *c*_0_, $c_{ii}^{\prime }=c_{0}^{\prime },$ $c_{0}<c_{0}^{\prime }$.
2. for all *i*, *j* ∈ *N*, *i* ≠ *j*, $c_{ij}^{\prime }=c_{ij}\leq c_{0}$

then, $C_{m}^{\prime }=C_{m}+\left(c_{0}^{\prime }-c_{0}\right).$ On the other hand, $d_{i}^{\prime }=c_{0}^{\prime }$ and *d*_*i*_ = *c*_0_, for all *i* ∈ *N*. So, as all claims are identical for all the individuals, the *CEL* rule allocates the same amount *B*/*n*, *B*′/*n* to each individual and

$$\begin{array}{c} \alpha_{i}^{ceq}\left(N_{\omega },C^{\prime }\right)=\frac{C_{m}^{\prime }}{n}=\frac{C_{m}}{n}+\frac{c_{0}^{\prime }-c_{0}}{n}=\alpha_{i}^{ceq}\left(N_{\omega },C\right)+\frac{c_{0}^{\prime }-c_{0}}{n}\;\forall i\in N \end{array}$$

which proves that this solution fulfills *equal share of extra-costs*.

**(7)** Example 2 shows that *α*^*ceq*^ does not fulfill *separability* nor *core selection*. Since *population monotonicity* implies *core selection*, our proposal does not fulfill *population monotonicity*.

**Example 2** *Let us consider the mcst problem defined by the following picture (arcs not depicted have a cost c*_*ij*_ = 2):

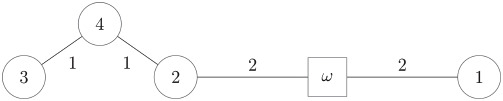


*There are several spanning trees with minimum cost C*_*m*_ = 6. *One of them is given by*:

$$\begin{array}{c} m(1)=\omega \;m(2)=\omega \;m(3)=4;\;m(4)=2. \end{array}$$

If we denote *S* = {1}, *T* = {2, 3, 4}, *m* = *m*^1^∪*m*^2^, where *m*^1^ and *m*^2^ are the minimum cost spanning trees in problems (*S*, **C**|_*S*_) and (*T*, **C**|_*T*_), respectively. On the other hand, $c_{ii}^{*}=2$, for all *i* ∈ *N*, and then *B* = 2 and $\alpha^{ceq}=(3/2,3/2,3/2,3/2)$. We observe that the *separability* property implies *α*_1_ = 2, so *α^ceq^*> does not fulfill this property. Also note that

$$\begin{array}{c} v\left(T\right)=4<\sum_{i\in T}\alpha_{i}^{ceq}=\frac{9}{2} \end{array}$$

so this proposal is not a core selection.

## Final remarks

We have proposed an egalitarian approach to the problem of sharing the common cost *C*_*m*_ of a *mcst*. One of the main features in our model is to ignore the “non-relevant” costs, considering only the cost of connecting the individual to the source, $c_{ii}^{*},$ and the total cost *C*_*m*_. Our first attempt to define an egalitarian allocation, the *α^ceq^*> solution is very simple to understand and to compute. It captures a *solidarity* approach in which the optimal cost (obtained throughout cooperation) is paid as equally as possible among the agents in the project. Nevertheless, it fails to be coalitionally stable, a crucial property whenever cooperation is necessary. Moreover, the egalitarian criteria will always ignore situations like the one in Example 2, where separability is not fulfilled.

To solve the core stability, we define the *β^ceq^* solution: the most egalitarian allocation in the core. We have shown that this solution coincides with the *Folk* solution in elementary problems. This fact allow us to reinterpret the *Folk* solution as the piece-wise linear extension of *β^ceq^* applied to elementary problems (the *Υ* solution).

Finally, an interesting scenario to extend (in future research) both the *Folk* solution and our egalitarian proposals is the *generalized minimum spanning tree problem*. In this problem, individuals are grouped into a number of predefined clusters, and the objective is to find a minimum-cost spanning tree of a subset of individuals which includes exactly one individual from each cluster (see, for instance, [20, 21]). As mentioned in those papers, “the generalized minimum spanning tree problem provides an attractive way of modeling various real world applications: in the field of telecommunications, in identifying the position of regional service centers such as stores, warehouses or distribution centers, agricultural settings, energy transportation, physics, etc.” Once the optimal spanning tree is obtained, as in our setting, its cost must be shared by the *agents* (clusters), and the allocation of each cluster must be shared by the individuals in this cluster.
